# Supplementary material for: Are carbon emissions trading and green financial instruments synergistic? -Comprehensive quantitative research based on content analysis
Source: PLoS One. 2024 Mar 7;19(3):e0298601. doi: 10.1371/journal.pone.0298601 (PMC10919601; doi:10.1371/journal.pone.0298601)
Supplement: S1 Data — (ZIP) [file pone.0298601.s001.zip › Data/地级市Z_F工作报告.zip!/║╙─╧/╨┬╧τ╩╨/└┤╫╘╣1⁄2╓┌║┼í╛┬φ┐╦╩2╛▌═°í┐_2004.docx]

各位代表：

　　现在，我代表市人民政府，向大会作工作报告，请予审议，并请各位政协委员和列席人员提出意见。

　一、过去五年工作回顾

　　1999年以来，本届政府在省委、省政府和市委领导下，在市人大、市政协监督、支持下，团结带领全市人民，与时俱进，真抓实干，奋力开拓创新，在全面建设小康社会道路上迈出坚实步伐。

　　综合经济实力明显增强。五年来，全市国内生产总值由1998年的256亿元增加到380.8亿元，年均增长8.9%。人均国内生产总值达到6948元，年均增长8%。规模以上工业增加值达到99.9亿元，年均增长11.9%。地方财政一般预算收入达到15.5亿元，年均增长4.8%；2003年财政支出35亿元，是1998年的2.2倍，年均增长17.2%。金融机构各项存款余额438.6亿元、各项贷款余额299.2亿元，分别比1998年增长83.1%和48.8%。社会消费品零售总额133亿元，年均增长7.8%。

　　经济结构进一步优化。一、二、三产比例调整为15.7∶50.1∶34.2，第二产业比重突破50%，比1998年提高10.3个百分点。优质专用小麦发展到315万亩，占73.4%；畜牧业产值占农业产值比重达到41.7%，比1998年提高10.9个百分点。全市实施工业结构调整项目336个，总投资68.5亿元，年均增长38.8%。实施高新技术产业化项目23个，总投资9.6亿元。新飞百万台空调生产线、白鹭化纤1.5万吨粘胶长丝、金龙铜业外翅片强化传热铜管、华兰生物年产150吨干灭病毒血浆、科隆公司5000吨高性能球形氢氧化镍、新乡火电厂2×13.5万千瓦机组等53个重点项目竣工投产。宝泉抽水蓄能电站4×30万千瓦机组、豫新电厂2×30万千瓦热电联产、白鹭化纤万吨粘胶长丝第二生产基地和平原、天瑞、孟电三大日产万吨新型干法水泥基地、五得利集团日产4000吨面粉生产线、新亚集团林浆纸一体化等27个投资亿元以上的重点项目开工建设。旅游服务设施完成投资3.4亿元，旅游综合收入达到4.5亿元，创建国家2A级景区5个。固定、移动电话用户分别达到105万和68万。中原沥青原料配送中心等现代物流项目和东方文化步行街等现代服务业项目正在建设。五年新增城镇人口30.9万，城镇化率达到30.2%，比1998年提高6个百分点，是改革开放以来城镇化速度最快的时期。

　　基础设施建设成效显著。累计完成全社会固定资产投资439.5亿元，年均增长22.2%。其中实施国债项目250个，完成投资26.4亿元。城市建设完成投资52亿元，其中2003年为16.5亿元，比1998年增长20.6倍。完成了体育休闲广场、卫河公园一期改造、六大出入市口道路、城市污雨水泵站、市区公厕新建和改造等13项重点基础设施项目；新建、改造了平原路、东干道、牧野路等52条（段）城区道路，其中新建道路24条（段）；实施了骆驼湾污水处理厂、市第五水厂、小尚庄污水处理厂、城市生活垃圾无害化处理场等8个国债项目；城市新区、科教园区和小店工业区已具雏形；人均绿地面积由1998年的2.9平方米增加到6.1平方米。累计完成公路投资50.1亿元，新建改建公路3992公里，其中新增干线公路里程392公里；新增高速公路42公里，通车里程达到80公里；新增682个村通油路。发电装机容量达到117万千瓦，比1998年增长93.4%；完成电网改造投资15.3亿元。完成大功、韩董庄、石头庄灌区节水改造和陈家院水库除险加固工程，天然文岩渠治理等工程正在实施。完成退耕还林34万亩，太行山绿化17.3万亩，通道绿化3257公里，森林覆盖率达到17%。

　　改革开放取得重大进展。政府机构改革顺利推进，市政府机构和行政编制分别减少28.8%、24.6%。列入全省统计范围的159户国有和市属集体工业企业，基本完成改制和按照改制预案进入实质性操作的122户，占76.7%。个体、私营企业发展迅速，非公有制经济占GDP的比重达到42%。完成农村土地经营权第二轮承包，农村税费改革取得阶段性成果，农民总体减负45.8%。财税、金融、流通、医疗保险和住房等改革稳步推进。对外开放取得明显成效，五年共引进外商投资企业76 家，实际利用外资2.2亿美元，外贸出口年均增长42.2%。

　　社会事业全面发展。取得省级以上科技成果56项，获授权专利1146项；科技对经济增长的贡献率达到39.4%。国家“细胞分化调控重点实验室”培育基地和新飞、华兰、金龙博士后科研工作站获准建立。“两基”教育目标提前实现，普通高中和职业教育发展迅速，中小学危房改造进展较快，河南师大、新乡医学院、职业技术师范学院、机电专科学校、师专等高校扩招工程多数竣工，高校招生规模比1998年增加3万人。加强环境保护，合理利用土地资源，连续5年实现耕地占补平衡。顺利完成市区区划调整，市区面积增加到346平方公里，人口达到87.7万人。市中心医院内科病房楼、人民医院病房楼、广电演播中心、平原省会旧址改造、防震抗震指挥中心等项目投入使用。人口自然增长率控制在7‰以内。军民共建和民兵预备役工作富有成效，荣获“双拥模范城”三连冠。体育、统计、审计、物价、外事、侨务、民族宗教、气象、人防、档案、史志等工作都取得新的成绩。

　　人民生活水平不断提高。城镇居民人均可支配收入年均增长4.4%，农民人均纯收入年均增长1.7%。城镇居民食品消费占消费总支出的比重（恩格尔系数）由41.5%下降到36%，农村居民由54%下降到45.6%，衣食住用行都有较大改善。在全省率先实行企业基本养老保险市级统筹，6.4万名国有企业下岗职工基本生活费和11万名企业离退休人员基本养老金按时足额发放，38.6万名城镇职工基本医疗得到保障，工伤、生育保险参保人数分别达到21万和17.8万人。建立城市居民最低生活保障制度，12.1万困难居民应保尽保。在全省首家全面启动新型农村合作医疗制度，使338万农民受益。帮助5.8万名下岗失业人员实现再就业。五年累计投入救灾款物6763万元，恢复重建住房3.1万间，救助灾民145万人次。“八七”扶贫攻坚计划基本完成，15万农村贫困人口实现温饱。深山区人畜吃水困难基本解决。

　　政治文明和精神文明建设进一步加强。“三讲”集中教育和“三个代表”学习教育活动成效显著。广泛开展“热爱新乡，增辉中原”、“道德规范进万家”等群众性文明创建活动，广大干部群众精神风貌发生新的变化。自觉接受人大法律监督和政协民主监督，认真办理人大代表建议和政协委员提案，五年累计办理2868件，办结率100%。省级园林城市、卫生城市创建成功。大力整顿规范市场经济秩序，优化发展环境。坚持从严治警，深入开展严打整治斗争，加强社会治安综合治理，切实做好信访工作，社会大局稳定。严肃查处各类违法违纪案件，纠正部门和行业不正之风，廉政建设和反腐败斗争取得阶段性成果。

　　服务型政府建设取得新成绩。规范和扩大政务公开，在全省率先实行市民旁听政府常务会议制度，建立重大决策听证和公示制度，免费发行《政府公报》。深化行政审批制度改革，市级审批事项由1572项减少到321项。推行首问负责制、服务承诺制和限时办结制，行政效率不断提高。推进政企分开、政事分开、事企分开，发展各类行业协会和中介组织。建立行政效能监察制度和“111”行政效能监察联动中心，政府工作作风明显转变，真抓实干的工作机制逐步形成。

　　2003年是不寻常的一年。全市人民万众一心，众志成城，取得抗击非典和洪涝灾害的重大胜利，国民经济实现速度和质量同步提高。国内生产总值增长13.5%，高于全国、全省平均水平。全社会固定资产投资增长52.9%，增幅居全省第二位。地方财政一般预算收入增长21.8%。规模以上工业增加值增长19.4%，实现利润增长31.4%。第三产业增加值增长9.6%，社会消费品零售总额增长11.2%。外贸出口增长49.7%，实际利用外资增长22.5%。城镇居民人均可支配收入和农民人均纯收入分别增长10.4%、5%。初步实现了经济全面提速和社会全面进步的目标。

　　我们深切地感到，本届政府工作的成绩，得益于历届政府打下的良好基础，凝聚着全市人民的辛勤劳动，包含着在新乡投资创业、工作学习的中外朋友们的贡献。在此，我代表市人民政府，向全市广大工人、农民、知识分子、干部、人民解放军指战员、武警官兵、公安民警以及各界人士致以崇高的敬意!向关心、支持我市现代化建设的香港特别行政区同胞、澳门特别行政区同胞、台湾同胞、海外侨胞和国际友人表示衷心的感谢!

　　各位代表，五年来，我们在实践中进一步深化了对新乡经济社会发展特点的认识，也得到了不少有益的启示。

　　（一）坚持用发展大目标统一思想，上下共谋发展。始终把发展作为第一要务，以“提速晋位”目标统一全市思想，激发干事创业热情，克服种种困难，不断冲破发展瓶颈，开创经济社会提速发展的新局面。

　　（二）坚持以项目统领经济工作全局，投资拉动发展。针对新乡大项目较少、经济发展后劲不足的状况，强力实施项目带动战略，把争取资金和项目建设摆在突出位置，固定资产投资跃居全省领先水平。

　　（三）坚持扩大对外开放，开放带动发展。着眼国际国内两个市场，增强开放意识，广泛开展对外合作，创新招商方式，拓宽引资领域，努力提高经济外向度。

　　（四）坚持建设服务型政府，环境促进发展。牢固树立执政为民理念，转变政府职能，推行政务公开，大幅度核减审批事项，提高办事效率，建立“责任、有序、高效”的工作机制，积极营造服务优、成本低、回报快、信用好的环境优势。

　　（五）坚持维护发展大局，团结保障发展。自觉维护班子团结、干群团结，以事业凝聚人心，致力于形成“心齐、气顺、风正、劲足”的工作局面，创造和维护同心同德干事业、一心一意谋发展的良好氛围，为经济社会全面发展提供保障。

　　在肯定成绩、总结经验的同时，我们也清醒地看到存在着很多困难和问题：一是城乡居民收入增长缓慢，人均GDP、财政一般预算收入、消费品零售总额还处于全省中下游。二是就业再就业和社会保障任务仍很艰巨，全市离退休职工和在职职工比例为1：2.6，远高于全省的1：4.3，财政包袱沉重。三是国企改革困难较多，全口径统计的711家国有、集体企业绝大多数未进行产权改革，大部分生产经营困难。四是利用外资规模小，对外开放程度低，发展环境仍需进一步改善。五是影响社会稳定的因素在一定范围内比较集中，由于阶段性和历史性遗留问题而引发的群体性事件时有发生。六是政府管理方式、工作作风等方面有待继续改进，反腐败工作任务还很艰巨。对于以上问题，特别是前进中涉及全局的倾向性和苗头性问题，我们将认真分析，区别对待，坚持用改革和发展的办法，着力加以解决。

二、今后五年政府工作的总体要求和奋斗目标

　　今后五年，是大力推进工业化、城镇化和农业现代化，为率先实现全面建设小康社会目标打基础、增速度的关键时期；是跨越两个五年规划，承前启后、继往开来的关键时期；是进一步深化改革、扩大开放，构建开放型社会，促进经济全面、协调、可持续发展的关键时期。

　　今后五年政府工作的总体要求是：以邓小平理论和“三个代表”重要思想为指导，全面贯彻党的十六大和十六届三中全会精神，坚持科学的发展观和“五个统筹”方针，以“推进经济跨越式发展、全面建设小康社会”为主线，深化改革，扩大开放，强化项目带动，加速工业化、城镇化、农业现代化和社会法制化进程，打造中原地区先进制造业基地、高素质人力资源培育基地和市场物流中心、现代农业示范中心，努力建设经济繁荣、人民富裕、环境优美、文明进步的中原城市群强市。

　　全市经济社会发展的主要预期目标是：国内生产总值年均增长11%以上；全社会固定资产投资年均增长20%以上；限额以上工业增加值年均增长13%以上；社会消费品零售总额年均增长10%以上；财政一般预算收入与国内生产总值同步增长；人口自然增长率控制在7‰以下；城镇登记失业率控制在4.5%左右。

　　――经过五年努力，基本形成与率先实现全面建设小康社会目标相适应的经济规模，实现综合实力的新跨越。国内生产总值力争达到700亿元，财政一般预算收入力争实现30亿元，三次产业比例大致为10∶55∶35，人均GDP力争突破11000元，实际利用外资和外贸出口分别达到1亿和4亿美元，非公有制经济总量占GDP比重达到70%左右，初步建成中原城市群经济强市。

　　――经过五年努力，基本形成农业现代化的框架体系，实现农村经济质量和效益的新跨越。基本完成以大型灌区续建配套和节水改造、六大林业工程建设为主导的农业生态体系，建成以优质小麦、优质水稻和绿色畜产品为主体的生产加工体系，完善农业质量标准、农民科技培训推广、农村信息化三大服务体系，基本实现农作物主要生产环节的机械化，初步建成中原城市群现代农业示范中心。

　　――经过五年努力，基本形成以高新技术产业为先导、七大特色产业为支撑的工业格局，实现工业经济竞争力的新跨越。基本形成电子电器、生物医药、环保电池及原料、化纤纺织、能源电力等五大强势产业基地和食品加工、汽车及零部件、起重机、造纸、医用卫材、振动机械、新型建材等七大特色产业，新飞电器、白鹭化纤、金龙铜业、安美电子、华亚钢铁、环宇电源、太行振动等向销售收入百亿元大型企业集团迈进，初步建成中原城市群先进制造业基地。

　　――经过五年努力，基本形成科学合理的城镇布局，实现城镇化进程的新跨越。构建起以中心城、卫星城和重点镇为主的市域城镇体系，各卫星城、重点镇基础设施及产业支撑的城市框架基本形成，全市城镇化水平达到40%以上，努力创造天蓝、地绿、水清、安全的人居环境。

　　――经过五年努力，基本形成教科文卫等社会事业统筹发展的良好局面，实现社会全面进步的新跨越。巩固提高基础教育，基本普及高中教育；加快发展高等教育、职业教育，规模分别达到11万人、10万人，初步建成服务新乡、辐射中原的高素质人力资源培育中心。健全知识产权保护体系，增强科技创新能力，科技进步对经济增长的贡献率超过40%。公共卫生体系更加健全。“牧野文化”品牌基本确立。

　　――经过五年努力，基本形成以人为本的社会保障机制，实现人民生活水平的新跨越。社会保障体系和社会救助制度更加健全，城乡弱势群体基本生活得到保障，就业更加充分。城镇居民人均可支配收入、农民人均纯收入分别超过9000元、3000元，人民生活更加富裕。

　　――经过五年努力，基本形成“三大文明”协调发展的新局面，实现社会文明程度的新跨越。依法治国和以德治国方略得到全面贯彻，以人为本，全心全意依靠群众、全心全意服务群众、全心全意造福群众的执政理念牢固树立，服务型政府的管理体制基本形成，以社会诚信体系为支撑的“知信用、守信用、用信用”、和谐信任的社会环境得以实现，科学、健康、文明的生活方式蔚然成风。

三、今后五年政府工作的主要任务

　　（一）突出特色产业、优势企业、产业集群、重工业发展四大重点，推进中原城市群先进制造业基地建设

　　把握工业发展规律，确立正确指导方针。一是坚持依靠技术的跨越实现工业的跨越发展，走新型工业化道路。积极发展对经济增长有突破性带动作用的高新技术产业；注重运用高新技术和先进适用技术改造提升传统产业；高度重视资源节约与综合利用和环境保护工作。二是适应我国经济从轻化工业向重化工业变革、以重型化为特征的新一轮大发展形势，坚持在继续发挥我市轻纺产业优势的基础上，着力推动重工业发展。三是依据工业的产业发展规律和市场竞争需要，坚持促进产业集聚发展，形成产业集群。积极推进高新技术开发区及西区、小店工业区、桥北新区、吴村重工业区、汽车及零部件工业园、新型电池产业园和各类特色园区建设，形成我市工业的强力增长极。

　　培育五大强势产业基地，发展七大特色产业。突出抓好以安彩信息产业园和新飞电子电器产品为主体的电子电器产品基地；以华兰、华星、新谊、拓新、佐今明为主体的生物与新医药制药基地；以环宇、金龙、安彩新能源、科隆为主体的环保电池及原料基地；以白鹭化纤、开元纺织、太阳石等为主体的化纤纺织基地；以新乡火电厂、宝山电厂、宝泉抽水蓄能电站等电力企业和辉县煤炭产业为主体的能源电力基地。重点发展汽车及零部件、食品加工等七大特色产业，逐步成长为在全省乃至全国占有重要地位的优势产业。

　　培育大型企业集团，提高工业整体竞争力。着力培育年销售收入10亿元以上和利税5000万元以上的企业50家，力争10家企业年销售收入超过50亿元，其中1―2家实现100亿元。

　　加大项目实施力度，壮大工业经济规模。规划实施1000万元以上的工业、能源项目325项，总投资约866亿元。确保安彩集团信息产业园、新飞网络计算机和税控机生产线、白鹭化纤年产6000吨氨纶、金龙铜业3万吨精密铜管、中新化工20万吨甲醇、新亚集团林浆纸一体化及平原、天瑞、孟电三大集团日产万吨新型干法水泥等265个总投资478亿元的重点项目竣工投产。开工建设白鹭化纤第二生产基地系列项目、金龙工业园系列项目、中新化工40万吨甲醇等60个总投资388亿元的重点项目。

　　（二）贯彻全市城镇化发展规划纲要，继续实施六大工程，推进城镇化进程

　　提升城市规划发展水平。根据《新乡市城镇化发展规划纲要》，完成城市总体规划、土地利用总体规划和市域城镇体系规划的修订编制工作。坚持市区中心城市建设为主导，长垣、原阳两个副中心城市和以市区为核心30分钟通勤圈内核心城市群为支撑的“一主两支”城市发展策略推进城镇发展。按照“一城三区（主城区和风泉、小店、小冀）、两级中心（市级行政中心、区级行政中心）、两个阶段、异质互动、有机增长（2015年前为主城区界限内集约增长，以后为界限内和三个区互动有机增长）”的思路推进市区扩张，提高中心城市的首位度，发挥带动作用。

　　继续实施城建交通六大工程。加快路网工程建设，完善市域高速公路网络体系，建成以市区为中心的城镇密集区30分钟通勤圈，县道和乡道分别达到二级、三级标准；加快环保工程建设，市区和县城污水及垃圾处理、燃气及热网工程等建成使用；加快园林绿化工程建设，以环城生态防护林和通道绿化为重点，建设绿树环绕、环境优美的生态城市；加快住宅工程建设，开发经济适用房，改造危旧房，实施康居工程；加快河渠工程建设，完成人民胜利渠、卫河和赵定排综合治理；加快大型公共设施工程建设，筛选、论证、建设一批城建精品。

　　落实推进城镇化的各项政策。支持县城和重点镇建设，完善基础设施和城市功能。完成市区和县（市）城镇规划区内的撤村建居工作。落实户籍管理、社会保障、农村土地流转等改革措施，为进城农民解除后顾之忧。

　　（三）大力发展县域经济，调整农村经济结构，推进中原城市群现代农业示范中心建设

　　加大对县域经济的扶持力度。县域经济发展与中心城市带动，是实现中原崛起、新乡振兴总体战略布局的两个重要方面，是基石和柱石的关系。要围绕富民强县两大目标，以工业化为主导、城镇化为支撑、农业现代化为基础，创新体制，扩大开放，营造环境，大力发展非公有制经济，培育发展特色支柱产业，推动县（市）综合实力显著增强。力争县域生产总值占全市的70%；各县（市）财政一般预算收入翻一番，达到16.5亿元，占全市的55%；县域二、三产业增加值在县域GDP中的比重达到80%；县域城镇化率达到30%；确保新乡县、辉县市在全省20强中位次明显前移，力争2―3个县（市）跨入全省发展速度“十快”行列，其他县（市）均要实现大幅度的晋位升级。扎实推进农村区域性经济中心建设，培育具有活力的经济增长点。

　　继续实施农村六大工程。围绕全国优质小麦基地建设，推进粮食优质化工程；以黄河滩区绿色养殖带、高产奶牛生产基地建设为重点，推进绿色畜牧业工程；实施环城防护林、沿山经济林和世行贷款项目，推进林业产业化工程；积极发展以无公害蔬菜、花卉、反季节瓜果等为主的生态农业，推进高效农业园区工程；健全劳务输出体系，强化对农民的就业培训和创业指导，推进劳务输出工程；加强农田水利设施和黄河滩区安全建设，推广“生态家园”沼气综合利用，推进农业基础设施工程。实施山区移民扶贫，从根本上解决贫困山区群众生活困难。

　　加快现代农业示范中心建设。以省农科院在原阳的5800亩高科技示范基地和敦煌种业基地为龙头，以现有高效农业园区为基础，促进农业生产单位的市场化兼并，提高农业技术含量、装备水平和综合效益。

　　促进非公有制企业发展。健全非公有制经济服务体系，引导其完善法人治理结构，兼并、扩张和对外合作，落实各项扶持政策，促其组织结构、经营团队、发展理念、产品设备的转型升级。

　　进一步深化农村改革。落实五年内取消农业税政策，完善农民负担管理机制，巩固农村税费改革成果。建立健全三大服务体系和农业风险保障、农村土地使用权流转两大机制。创新农村经营体制，培育各类经济合作组织。深化粮食流通体制、农村科技推广体制改革及农村信用社、供销社改革。建立健全农村低保、养老保险和新型合作医疗等农村社保体系。

　　（四）打造服务新乡辐射中原的市场物流中心，推进服务业现代化进程

　　壮大提升商贸流通业、房地产与市政服务业、金融保险业、教育卫生服务业四个传统行业，大力发展现代物流业、信息服务业、文化旅游业、中介服务业、社区服务业五个新兴行业。加快物流资源整合，逐步形成以综合物流为基础、以城市配送为支撑的两大物流体系。鼓励商贸流通企业对外合作，促进业态升级。以城镇社区基础设施和服务体系建设为载体，做大做强社区服务业。推进太行山旅游带、卫河文化旅游带和沿黄生态旅游带建设，注重旅游产品开发和推介。加强电话网、电视网、互联网和政务信息化建设。完善住房、汽车、教育等信贷政策，促进消费结构升级。

　　（五）培育人才、技术优势，统筹发展各项社会事业

　　把教育放在优先发展的战略地位。增加农村教育投入，完善“以县为主”的农村义务教育管理体制，农村中小学危房占校舍总面积的比例控制到0.5%以下。优化中小学布局，加强示范性高中建设，促进优质教育资源扩张发展。加快科教园区建设，支持驻市高校扩大规模，大力发展职业教育。认真做好人才强市工作，努力营造各类人才脱颖而出、人尽其才的良好环境。

　　健全完善科研创新体系。推进企业与高校、科研机构开展科技合作，鼓励大型企业建立研发机构，成为科技创新和产业化的主体。充分发挥企业博士后科研工作站和省级工程研究中心的作用，形成一批具有自主知识产权的科研成果。

　　推进文化、卫生、体育等社会事业发展。加快城乡文化体育等公共设施建设。加强疾病信息网络、疾病预防控制和医疗救治等公共卫生体系建设，基本建成市、县、乡三级疾病预防控制体系。深入开展全民健身和体育进社区活动。

　　坚持走可持续发展的道路。加大环保基础设施建设和环境综合治理力度，下决心关闭“五小”企业，加快造纸、水泥等行业产业产品和组织结构调整。实施地下水保护行动计划。实行最严格的耕地保护制度，抓好土地开发整理，优化配置土地资源。积极做好老龄工作。落实人口与计划生育目标责任制，维持低生育水平，提高出生人口素质。

　　加强精神文明建设。贯彻实施《公民道德建设实施纲要》，积极开展群众性精神文明创建活动，倡导文明健康的社会风尚和生活方式。加强国防教育，支持军队建设，做好民兵预备役工作，巩固发展双拥成果，争创“双拥模范城”四连冠。

　　（七）进一步深化改革、扩大开放，为发展提供强大动力

　　深化各项改革。扎实推进国有、集体企业产权制度改革，实现国有资本有序退出。加速国有资产管理体制改革，建立国有资本经营预算制度和企业经营业绩考核体系，确保国有资产保值增值。加快投融资体制改革，建立责、权、利明确的投融资风险机制，推行政府性投资代建制和非政府性投资备案制。大力培育资本市场，积极推动金企合作，利用金融租赁、债券、信托等融资工具扶持企业发展。推进财税管理体制改革，建立健全公共财政体系。强化国债资金监督管理，提高资金使用效益。

　　大力发展开放型经济。建立完善组织体系、评价体系和责任追究制度，瞄准“长三角”、“珠三角”、环渤海经济圈和海外，开展长期定向招商。推行招商引资目标责任制、承诺上岗制、末位淘汰制。深化外经贸体制改革，引导外贸出口从收购制向代理制转变。落实生产企业自营出口免、抵、退税政策。积极支持中小企业和非公有制企业出口。培育壮大出口潜力大的纺织、医药、机电等主导产品，扩大高新技术和服务贸易出口。

　　深入开展信用城市建设。以社会信用制度建设为核心，建立健全失信惩戒和守信受益机制。从培育市场、促进发展入手，以建立个人和企业信用联合征信系统为重点，建立健全信用服务体系。以增强信用意识为重点，全力推进社会诚信活动，营造和谐信任的社会环境。

　　进一步优化发展环境。整顿和规范市场经济秩序，依法打击制假售假、欺行霸市、商业欺诈、偷逃骗税等违法犯罪行为。健全产品质量监管机制。加强安全生产和监察，遏制重大事故发生。整治企业周边环境，严肃查处“四乱”行为。完成“五城联创”目标。健全治安防控网络，保持严打高压态势，争创“社会治安综合治理先进市”。坚决打击“法轮功”等邪教组织非法活动。切实做好信访工作，维护社会稳定。

　　（八）构建就业再就业长效机制，健全社会保障体系

　　实施积极的就业政策。坚持集中力量安排下岗失业人员再就业与构建保证充分就业的长效机制相结合，坚持加快发展经济增加就业与深入挖潜开发就业岗位并重，坚持将促进就业的政府责任向政府主导的社会责任转变。以街道、社区劳动保障平台建设为载体，逐步建立统一的劳动力市场、完善的技能培训网络和健全的就业服务体系。加大再就业资金投入和援助力度，落实优惠政策，改善就业和创业环境。

　　做好“两个确保”和“低保”工作。强化社会保险基金清欠征缴，完善养老保险市级统筹运行机制，推进国企下岗职工基本生活保障向失业保险并轨和企业退休人员管理服务社会化。区别“低保”群体不同情况实行差别化保障，逐步提高低保标准。完善社会救助制度，帮助城镇特困家庭解决就医看病、子女入学等实际困难。

四、2004年政府重点工作

　　2004年是新一届政府履行职责的第一年，各项工作都要开好头、起好步，努力实现国内生产总值增长12%，全社会固定资产投资增长30%，地方财政一般预算收入与国内生产总值同步增长。今年要重点抓好九项工作：

　　（一）以招商引资为突破口，全面提高对外开放水平

　　健全四个招商机构，完善各项引资政策，全年合同利用外资、实际利用外资、利用市外资金分别突破1亿美元、6000万美元、30亿元人民币。全市拥有进出口经营权的企业达到400家，外贸进出口突破3亿美元。争取2家企业取得对外劳务经营权，外派海员600人次。

　　（二）继续实施项目带动战略，保持投资快速增长

　　实施投资千万元以上项目396项，其中亿元以上项目108个，计划总投资612亿元，年度投资150亿元。确保白鹭化纤5000吨粘胶长丝和3000吨氨纶、五得利公司日产2000吨面粉生产线、平原水泥厂日产5000吨新型干法水泥、华亚钢铁60万吨钢材等163项总投资151亿元的项目竣工投产。加快宝泉4×30万千瓦抽水蓄能电站、新亚集团林浆纸一体化、华兰公司浓缩物蛋白胶血浆血糖等244个总投资319亿元的续建项目建设。开工建设宝山电厂2×60万千瓦机组、渠东电厂2×30万千瓦热电联产、环宇集团年产6000吨锂离子电池材料、豫新机械厂大排量汽车空调压缩机生产线、娃哈哈方便面等152个总投资293亿元的项目。抓住国家发行1100亿元国债的机遇，争取更多的项目和资金。

　　（三）多策并举，确保农业增产和农民增收

　　贯彻中央、省农村工作会议和省县域经济工作会议精神，扩大县（市）经济社会管理权限，落实各类扶持政策。切实落实对农民承诺的56件实事，兑现农业税降低3个百分点、对粮食主产区农民直接补贴等扶持政策，实现农民人均减负增收54.2元。大力发展优质小麦、蔬菜、中药材种植和各类养殖小区。完成以环城生态防护林建设为重点的林业工程。抓好正面水库除险加固、引黄灌区节水改造、农村安全饮水等基础设施建设。新建农村户用沼气池10万个。加大劳务输出工作力度，增加农民务工收入。实现3.2万贫困人口稳定脱贫。发展农村经济合作组织，农民经纪人总数达到16万人。重点培育15家年销售收入超5亿元的非公有制龙头企业。争取非公有制经济增加值增长15%，税金增长13%。

　　（四）强力推进国企改革，加大工业运行协调力度

　　基本完成列入省统计范围的国企改革任务，通过借助外力、出资买断、破产重组等多种形式，推进企业产权制度改革。实现华兰公司股票上市，完成金龙铜业、环宇公司上市准备工作，推进黄河林业、太行振动、科隆电器上市辅导工作，争取1―2户企业进入上市辅导期。加强工业运行协调服务，保证资金和电力、油料等生产资料供应，提高工业经济效益。

　　（五）加快基础设施建设，完善城镇功能

　　修订编制城市总体规划、土地利用总体规划、8个县城及重点镇总体规划。完成市委、市政府迁址工作。建设新区C、D区住宅，加快完善新区路网和供排水管网，开工建设新区学校、医院等基础设施。新登记城镇人口10.6万人，城镇化率提高1.7个百分点。实施牧野公园等一批城市精品项目。建成京珠高速新乡至郑州段和原阳至新庄段高速公路；开工建设济东高速新乡段。完成三原线新乡―原阳等13条241公里干线公路和县道二级公路续建项目；开工建设S301原武―黄河桥北段等18条489公里干线公路和县乡公路新（改）建项目，实现平原地区行政村“村村通油路”目标；完成107国道互通式立交桥和市区到周边县（市）区快速路建设。新（改）建牧野路、平原路等21条（段）城市道路。续建小尚庄污水处理厂、垃圾处理场等一批环保工程。完成国家卫生城市和省级文明城市先进单位创建任务，申报国家园林城市、优秀旅游城市自测达标。

　　（六）大力发展现代服务业，推进区域物流中心建设

　　着力实施中原沥青原料配送中心等7个重点物流项目，培育小店物流港、邮政物流中心、新乡铁路货运中心三大物流集团，发展豫北汽车城、豫北建材大市场等八大批发交易和配送中心，筹划实施桥北新区物流园区、南环市场群、种子物流配送项目。完成社区服务中心建设，发展“6612390”加盟企业100家、社区服务网点200家。创建4A级旅游景区1―2家，3A级景区3―4家，实现旅游综合收入增加50%以上。

　　（七）深化财税体制改革，积极培育资本市场

　　强化税收征管，加强税源监控，努力使财政收入规模与经济发展相适应。认真落实所得税收入分享改革，加大对县乡财政的支持力度。完善财政涉农政策，增加对农村基础设施的投入。加强地方金融企业的监管工作，确保不良资产清收率达到30%以上。建立资本市场发展促进机构，加快资本市场培育，为经济发展提供更有效的服务。

　　（八）健全社会保障体系，帮扶弱势群体

　　完善企业养老保险市级统筹运行机制，加大清欠、扩面工作力度。落实“分类施保”的低保政策，实现动态管理下的应保尽保。开发、购买公益性岗位5.5万个，重点安置1万名“4050”人员就业。免费培训复员转业军人、城镇新增劳动力、下岗失业人员和农村富余劳动力2万人。确保200户无房户享受廉租房补助，9000人享受医疗、教育、法律援助等服务。

　　（九）加快发展教科文卫等社会事业，改善人民群众生活

　　抓好市一中等学校新校区和新区中小学建设。完成中小学D级危房改造任务。继续支持师专、平大、教育学院联合升本和机电专科学校升本，高校招生规模达到6.5万人。组建市职教中心和职业技术培训学院。加快党校（行政学院）建设。争取企业新建1―2家博士后科研工作站。加快中心医院外科病房楼、人民医院综合病房楼和8个县级传染病区、11个乡镇中心卫生院、30个社区卫生服务站等项目建设。完善农村新型合作医疗制度运行机制，扩大覆盖面。繁荣文化艺术、广播电视和新闻事业。举办市第八届体育运动会。切实搞好经济普查。认真搞好粮食清查挂帐审计和综合财力专项审计调查。严格保护耕地，加快土地开发整理。强化环境综合整治及秸杆综合利用，力争出境断面水质达标和大气环境质量根本改善。人口自然增长率控制在7‰以内。启动“双拥模范城”四连冠创建工作。加大社会治安综合治理力度，高度重视公安工作，妥善处理群众来信来访，维护正常社会秩序和社会稳定。认真做好防震减灾、物价、民族宗教、外事、侨务、档案、史志、文物、气象、人防等工作。

五、以提高行政能力和管理水平为重点

　　努力建设人民满意的政府

　　全市各级政府肩负着发展的使命，承载着人民的重托，面对新的形势和任务，必须改革不合时宜的管理体制和行政方式，提高行政能力和管理水平，以人为本，建设责任、法治、服务型政府。

　　推进政府职能转变，提高依法行政水平。认真贯彻《行政许可法》，按照法定权限和程序行使权力、履行职责。继续清理、规范审批事项，完善审批首席代表制、并联审批制和项目代理制，建立网上申报、审批新机制。强化政府社会管理和公共服务职能，积极推进事业单位机构和人事制度改革，大力发展、规范行业协会及社会中介组织，促进政企、政事、事企分开。做好新一轮行政机构改革和区划调整后机构及职能部门调整工作，严格一级对一级负责、一级支持一级的行政秩序，加强政府部门间的协作配合。强化目标管理考核和行政督查，推进“111”效能监察网络建设，确保政令畅通。完善市民旁听政府常务会议、重大经济社会问题听证和公示制度，健全政府新闻发言人制度，做好《政府公报》发行工作。

　　树立正确的政绩观，坚持科学的发展观。坚持执政为民，全心全意为人民服务，真正做到思想上尊重群众，感情上贴近群众，行动上深入群众。凡是得民心、顺民意的事情，都要竭尽全力去做，既要锦上添花，更要雪中送炭，真正把群众的愿望和呼声作为政府工作的出发点和落脚点，满腔热情地为人民群众多办好事实事。坚持求真务实，办事情、做决策，都要从实际出发，按客观规律办事，既要积极进取，又要量力而行，不急功近利，不虚报浮夸，不盲目攀比，不追求虚假繁荣。要以当地经济发展质量有没有提高、综合实力有没有增强、外来投资有没有增加、城乡人民生活是否得到改善等为主要标准，改进经济工作的评价和考核办法，促进资源永续利用和环境不断改善，决不能吃祖宗饭、断子孙路。

　　加强廉政建设，自觉接受人民监督。政府的一切权力都是人民赋予的，必须对人民负责，为人民谋利益，接受人民监督。只有人民监督政府，政府才不会懈怠。各级政府要自觉接受人大及其常委会的依法监督和政协的民主监督，认真办理人大建议和政协提案，广泛听取各民主党派的意见。要接受新闻舆论和社会公众监督，加强政府系统内部监督。全面落实党风廉政建设责任制，纠正部门和行业不正之风，严肃查处损害群众利益的行为，把好“制权、管钱、用人”关，努力从体制、机制上铲除腐败滋生和蔓延的土壤。

　　全体政府工作人员特别是各级领导干部务必做到政治坚定、业务精通、清正廉洁、作风优良。任何情况下都要自觉维护党和政府的形象，维护新乡的形象，不争权力大小，不争角色轻重，不争功过是非。要奋发有为，不要怨天尤人；要敢闯敢冒，不要亦步亦趋；要埋头苦干，不要清议空谈；要求真务实，不要浮躁作秀。我们肩负全面建设小康社会、实现中原崛起的重任，必须以只争朝夕的精神拼搏进取，以锲而不舍的精神团结奋进，谱写新乡经济跨越式发展的新篇章。

　　各位代表，新一届政府即将和全市人民一道，踏上新的征程，让我们紧密团结在以胡锦涛同志为总书记的党中央周围，高举邓小平理论伟大旗帜，全面贯彻“三个代表”重要思想，同心同德，励精图治，开拓前进，扎实工作，为把新乡建设成中原城市群强市而奋斗！
